# Bidirectional Mendelian randomization study of insulin-related traits and risk of ovarian cancer

**DOI:** 10.3389/fendo.2023.1131767

**Published:** 2023-03-01

**Authors:** Xinghao Wang, Jing Sun, Jia Li, Linkun Cai, Qian Chen, Yiling Wang, Zhenghan Yang, Wenjuan Liu, Han Lv, Zhenchang Wang

**Affiliations:** ^1^ Department of Radiology, Beijing Friendship Hospital, Capital Medical University, Beijing, China; ^2^ School of Biological Science and Medical Engineering, Beihang University, Beijing, China

**Keywords:** ovarian cancer, Mendelian randomization, insulin, insulin secretion rate, endocrine marker

## Abstract

**Background:**

It is well known that the occurrence and development of ovarian cancer are closely related to the patient’s weight and various endocrine factors in the body.

**Aim:**

Mendelian randomization (MR) was used to analyze the bidirectional relationship between insulin related characteristics and ovarian cancer.

**Methods:**

The data on insulin related characteristics are from up to 5567 diabetes free patients from 10 studies, mainly including fasting insulin level, insulin secretion rate, peak insulin response, etc. For ovarian cancer, UK Biobank data just updated in 2021 was selected, of which the relevant gene data was from 199741 Europeans. Mendelian randomization method was selected, with inverse variance weighting (IVW) as the main estimation, while MR Pleiotropy, MR Egger, weighted median and other methods were used to detect the heterogeneity of data and whether there was multi validity affecting conclusions.

**Results:**

Among all insulin related indicators (fasting insulin level, insulin secretion rate, peak insulin response), the insulin secretion rate was selected to have a causal relationship with the occurrence of ovarian cancer (IVW, P < 0.05), that is, the risk of ovarian cancer increased with the decrease of insulin secretion rate. At the same time, we tested the heterogeneity and polymorphism of this indicator, and the results were non-existent, which ensured the accuracy of the analysis results. Reverse causal analysis showed that there was no causal effect between the two (P>0.05).

**Conclusion:**

The impairment of the insulin secretion rate has a causal effect on the risk of ovarian cancer, which was confirmed by Mendel randomization. This suggests that the human glucose metabolism cycle represented by insulin secretion plays an important role in the pathogenesis of ovarian cancer, which provides a new idea for preventing the release of ovarian cancer.

## 1 Introduction

Ovarian cancer is a kind of cancer that occurs in female ovarian tissue. There are many pathological subtypes, among which high-grade serous ovarian cancer (1) is the most common. In developed countries, ovarian cancer is the main cause of death among all gynecological cancers (2). Due to the lack of specific signs and symptoms at the early stages of the disease, ovarian cancer is usually found in late stage, with extensive peritoneal (Phase III) or extraperitoneal (Phase IV) spread. Tumor reduction surgery and platinum and taxane drug chemotherapy could make 75% of patients achieve clinical remission. At present, the 5-year survival rate of ovarian cancer patients is roughly less than 30% (3–5). There are many risk factors for ovarian cancer, including age, reproductive history, changeable lifestyle factors, family history and gene mutation (6).

Insulin (7) is the main regulator of glucose, lipid and protein metabolism. When oral glucose load or mixed meal is ingested, plasma glucose concentration increases, and islets of β Cells are stimulated to secrete insulin. Insulin can inhibit the production of endogenous glucose (the main target organ is the liver), stimulate the uptake and storage of glucose by muscle, liver and fat cells, and inhibit the decomposition of fat, leading to a decrease in plasma free fatty acids concentration (8), which helps to inhibit the production of glucose in the liver and increase the uptake of glucose in the muscle, and can relax muscle vessels, which helps to enhance muscle glucose disposal.

The incidence rate of various cancers is higher in patients with insulin secretion disorder (especially in patients with type 2 diabetes). Many studies and observations in the field of overseas studies have confirmed this view. It is reported that among patients with type 2 diabetes, the relative risk of endometrial cancer, liver cancer and pancreatic cancer is more than 2 times, while the relative risk of bladder cancer, breast cancer and colorectal cancer is as high as 1.5 times (9–12). In addition to the increase in incidence rate, the overall mortality rate of diabetes patients when diagnosed with cancer is higher (13) than that of the non-disease group. Systematic reviews (14, 15) could suggest that overweight people have a higher risk of ovarian cancer, and the risk of ovarian cancer increases with obesity. The increase and abnormality of obesity or body mass index often could lead to the disorder of endocrine system in the human body, such as insulin resistance, estrogen level change and other characteristics, which are factors that cannot be ignored in the role of obesity factors in weight related cancer.

Therefore, it is important to understand the hormone specific relationship between metabolism and cancer (16). In this paper, bidirectional mendelian randomization analysis was used to confirm the causal relationship between insulin related characteristics and ovarian cancer risk.

## 2 Materials and methods

### 2.1 GWAS statistics of insulin-related traits

This study included six insulin related indicators from three studies, including Fasting blood insulin, Fasting blood insulin adjusted for BMI, Insulin secret rate, Peak insulin response, Acute insulin response and Insulin disposition index. The specific description of relevant data can be shown in **Table 1**.

**Table 1:** Description of relevant GWAS data.

Traits	Population	Sample size	Year	Number of SNPs
Fasting blood insulin	European	51,750	2012	2,598,774
Fasting blood insulin adjusted for BMI	European	30,825	2015	103,869
Insulin secretion rate	European	527	2017	6,919,421
Peak insulin response	European	2,337	2017	9,694,532
Acute insulin response	European	2,087	2017	9,663,724
Insulin disposition index	Hispanic or Latin American	2,345	2017	9,652,444

#### 2.1.1 Fasting blood insulin 

The GWAS data (17) came from Genome wide association studies for fast glucose (FG) and fast insulin (FI), which analyzed the exon array data of 33231 non-diabetes patients of European origin. The data and SNP of fasting insulin came from this study.

#### 2.1.2 Fasting blood insulin adjusted for BMI

The data of this indicator (18) came from a study of “Genome wide method considering body mass index determines genetic variation affecting fasting blood glucose characteristics and insulin resistance”, which includes 96496 non diabetes patients. The fasting insulin data here were adjusted by body mass index.

#### 2.1.3 Insulin secretion rate

This study explored the genome-wide association study based on IVGTT’s first phase insulin secretion measurement, which refined the potential physiology of type 2 diabetes variation. Insulin secretion rate (ISR) is the estimated insulin secretion rate (ISR) (19) based on the measured serum C-peptide concentration at 0, 2, 4, 6, 8 and 0, 2, 3, 4, 5, 6, 7, 8, 10, 12, 14, 16 and 19 (FAMILY) minutes. ISEC software (20) is used to calculate the secretion rate according to predefined C-peptide kinetic parameters, including each person’s weight, height, age Gender and clinical status (glucose tolerance and obesity status) were determined in a population-based study (21). The ISR provides an estimate of the rate of insulin secretion before hepatic insulin clearance.

#### 2.1.4 Peak insulin response

Peak insulin response was measured as peak insulin minus baseline insulin. Determine the peak insulin time point of each study according to the time point when the average insulin value of all individuals is the highest.

#### 2.1.5 Acute insulin response

The acute insulin response (AIR) was measured as the incremental area under the insulin curve in the first 10 minutes. Or if the 10 minutes measurement is not available, the minimum insulin value at 0, 2, 4, 6 and 8 minutes during the IVGTT using the trapezoidal equation during the first 8 minutes. Incremental insulin was calculated by subtracting fasting insulin levels.

#### 2.1.6 Insulin disposition index

Insulin disposition index was calculated as the product of AIR, and insulin sensitivity index was calculated by the MINMOD (22), which took into account the level of background insulin resistance.

### 2.2 GWAS statistics of ovarian cancer

Through IEU Open GWAS (MR Base) (23) public database(https://gwas.mrcieu.ac.uk/)to retrieve and obtain data on ovarian cancer. The data of ovarian cancer patients are from UK Biobank. According to the data, the latest update is 2021, which includes 9822229 SNPs from 199741 Europeans. The classification of data is binary data, that is, whether ovarian cancer has occurred. The website shows that 1218 patients were included, while 198523 patients were included in the control group.

### 2.3 Mendelian randomization statistical analysis

Two-sample bidirectional MR was used to test the causal relationship between insulin-related traits and tumors. In order to determine whether insulin-related traits could be a risk factor for various tumors, we first selected closely related SNPs from ovarian cancers’ GWAS results. In this process, insulin-related traits acted as exposure and ovarian cancer occurs as a result. In order to verify whether ovarian tumors cause insulin-related traits, SNPs related to various tumors are selected as the instrumental variable in the reverse MR analysis process, with ovarian tumors as the exposure, and insulin-related traits as the result.

Three different MR methods, including inverse variance weighted random effects (IVW), MR Egger and weighted median, were used to evaluate heterogeneity and test multiple effects. SNPs and outliers of insulin related traits identified by MR-PRESSO were removed. In the face of Mendelian randomization, IVW was used as the main analysis method, which was widely accepted. The threshold for screening exposure variables was 10^-6. MR Egger (24) was often a test that allows all genetic variations to have pleiotropic effects, but requires pleiotropic effects to be independent of the exposure association between variations. For Mendelian randomization pleiotropy test, MR Egger intercept test and retention analysis were used to further evaluate the level pleiotropy. Cochran’s Q test was implemented in each MR analysis to detect data heterogeneity between exposure and outcome, which was an important indicator affecting the reliability of final results. In the final visualization part, the funnel chart was used to evaluate the possible directional pleiotropy, similar to the evaluation of publication bias in meta-analysis, and also to observe the data distribution. The forest map is used to show the results of each SNP and the final MR, which was a convenient and intuitive method for visualizing the results.

All bidirectional mendelian randomization statistical analysis and data visualization used “TwoSampleMR” (https://github.com/MRCIEU/TwoSampleMR) in R software version 4.1.1. RStudio (https://posit.co/products/open-source/rstudio/) was used as a platform tool for opening and analysis, which was an integrated development environment for R and Python. It included a console, syntax highlighting editor that supports direct code execution, and tools for drawing, history, debugging, and workspace management. Bilateral P value less than 0.05 was considered statistically significant.

## 3 Results

Six insulin related indicators, including Fasting blood insulin, Fasting blood insulin adjusted for BMI, Insulin secret rate, Peak insulin response, Acute insulin response and Insulin disposition index, went Two-sample bidirectional MR with ovarian cancer. We conducted a total of 12 statistical tests in 6 groups.

As shown in **Table 2**, we summarize all positive MR results into this table. The insulin secretion rate was statistically significant (IVW, p<0.05).

**Table 2:** Description of MR result.

Traits	IVW-derived P value	OR (95% Confidence intervals)	Cochran’s Q-derived P value	MR-Egger intercept-derived P value
Fasting blood insulin	0.62030831	0.9976788 (0.9885484, 1.0068936)	0.2304561	0.02442566
Fasting blood insulin adjusted for BMI	0.4649689	0.9965644 (0.9874069, 1.005807)	0.4126454	0.9639076
Insulin secretion rate	0.0179683	0.9991305 (0.9984108, 0.9998507)	0.8227378	0.4466693
Peak insulin response	0.6999933	1.000156 (0.9993622, 1.000951)	0.5413675	0.9516094
Acute insulin response	0.4477386	0.9996450 (0.9987289, 1.000562)	0.8945821	0.6796435
Insulin disposition index	0.4849515	0.9996270 (0.9985809, 1.000674)	0.11898331	0.6133764

On the contrary, the other five insulin related indicators (Fasting blood insulin, Fasting blood insulin adjusted for BMI, Peak insulin response, Acute insulin response and Insulin disposition index) did not show any correlation with the risk of ovarian cancer (**Supplementary Figures S1**-**S5**).

**Figure 1:**
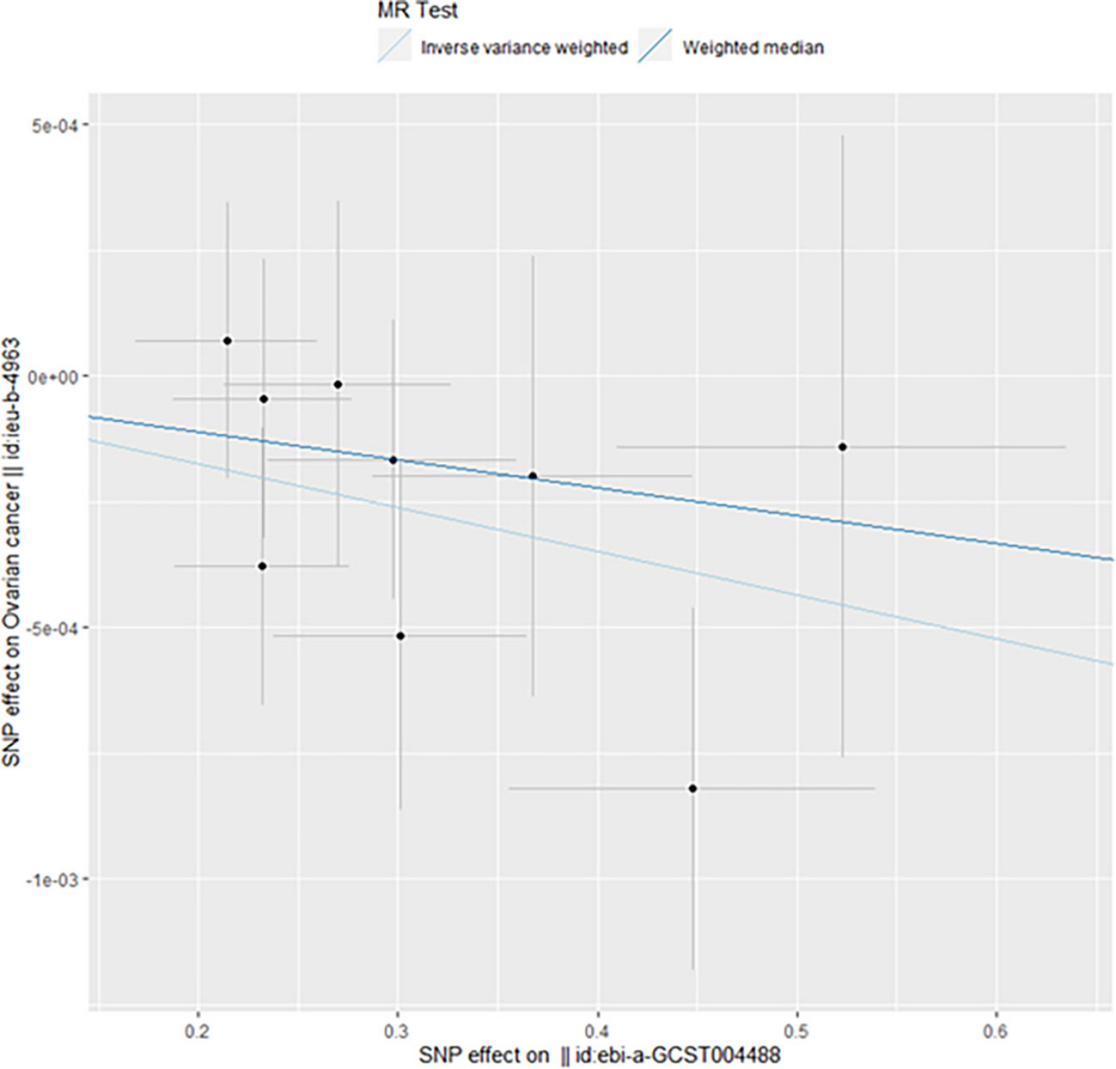
Mendelian randomization results of the association of the insulin secretion rate on ovarian cancer (Forward).

**Figure 2:**
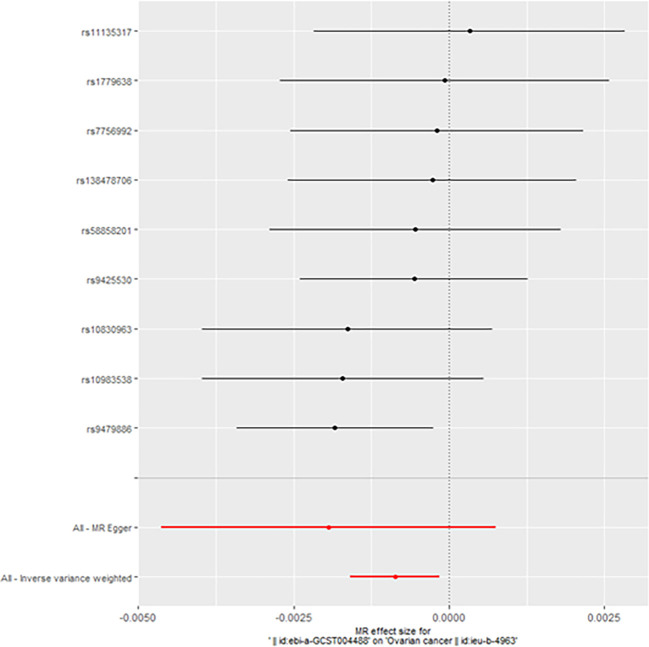
The pleiotropy test of MR analysis (Forward).

**Figure 3:**
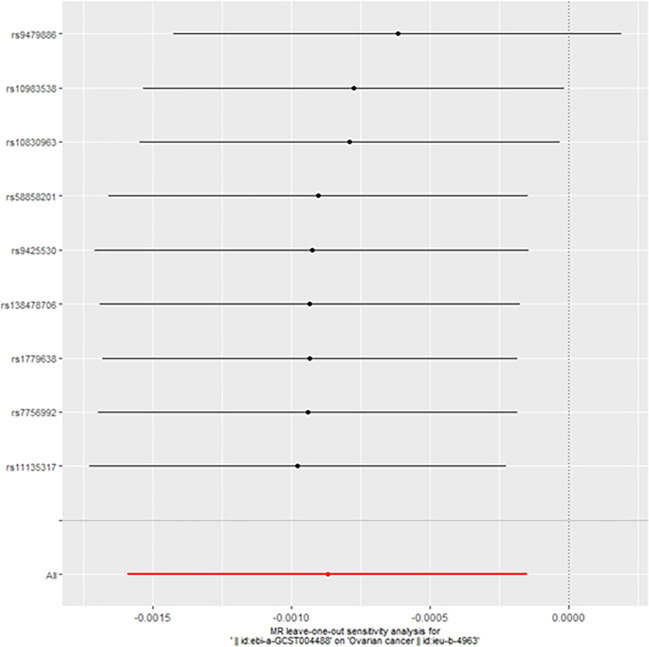
The retention analysis of the SNPs (Forward).

When taking insulin secretion rate as the exposure factor, we found that impaired insulin secretion was associated with an increased risk of ovarian cancer (OR 0.9991305 (0.9984108, 0.9998507), p=0.017968, **Figure 1**), which was confirmed in the positive MR analysis (**Figure 2**). There were 9 SNPs related to the above results (rs10830963, rs10983538, rs11135317, rs138478706, rs1779638, rs58858201, rs7756992, rs9425530, rs9479886), and the details were shown in **S-Table 1** of **Supplementary Materials**. For the pleiotropy test of MR analysis, no obvious pleiotropy was found (p>0.05, **Table 2**). The retention analysis of the above results shows that all SNPs are generally stable (**Figure 3**), and the funnel plot did not show significant heterogeneity (**Supplementary Figure S6**).

**Figure 4:**
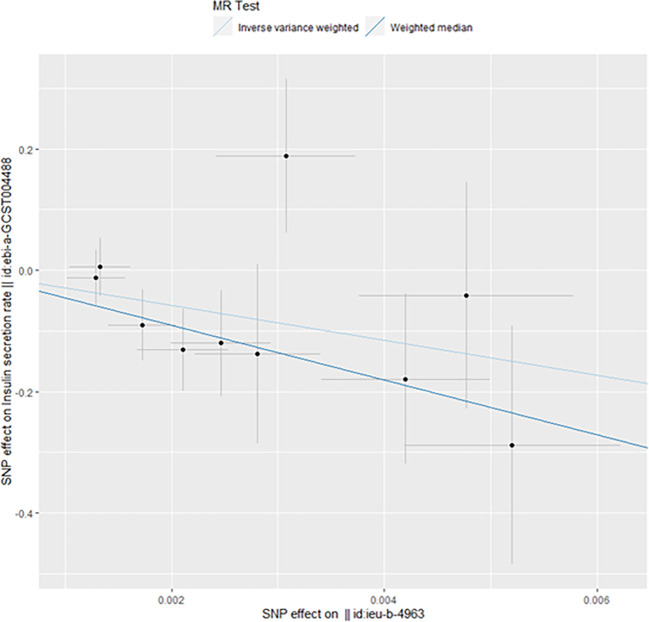
Mendelian randomization results of the association of ovarian cancer on the insulin secretion rate (Reverse).

**Figure 5:**
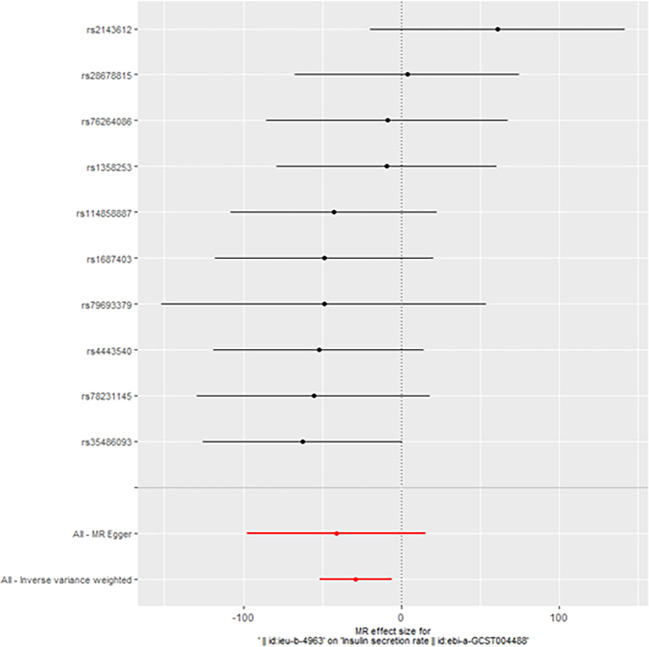
The pleiotropy test of MR analysis (Reverse).

**Figure 6:**
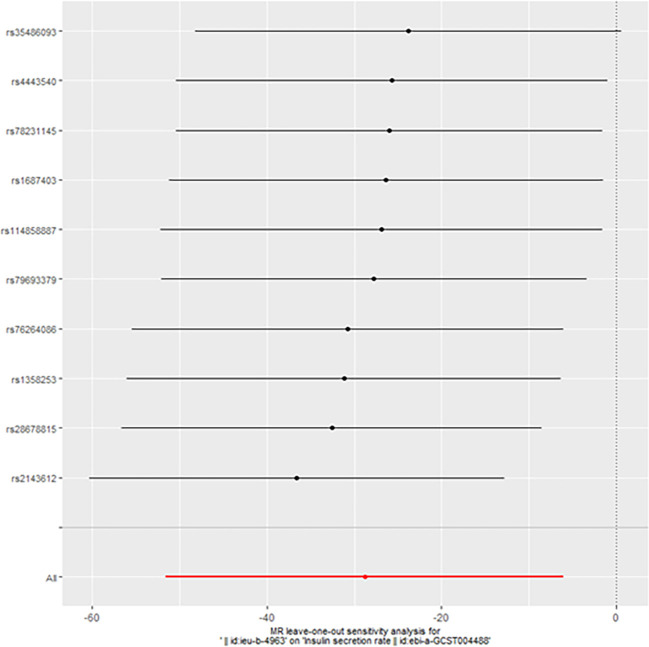
The retention analysis of the SNPs (Reverse).

However, we still got statistically significant results (IVW, p<0.05) when we performed reverse MR analysis on the insulin secret rate. That was to say, with ovarian cancer as the exposure factor and the insulin secret rate as the outcome variable, we still got the causal relationship of the above two parties (OR 3.092427e-13 (3.816945e-23, 2.505434e-03), p=0.013, **Figure 4**), which was confirmed in the inversive MR analysis (**Figure 5**). The above results indicate that ovarian cancer had a causal relationship with human insulin secret rate. In order to test the reliability of the above results, we conducted Cochran’s Q test (p=0.4528141) and pleiotropy test (p=0.6568936). However, these test results indicate that the above results do not have the pleiotropy and heterogeneity of imaging conclusions. There were 10 SNPs related to the above results (rs114858887, rs1358253, rs1687403, rs2143612, rs28678815, rs35486093, rs4443540, rs76264086, rs78231145, rs79693379), and the details were shown in **S-Table 2** of **Supplementary Materials**. The retention analysis of the above results showed that all SNPs were generally stable (**Figure 6**), and the funnel plot did not show significant heterogeneity (**Supplementary Figure S7**).

## 4 Discussion

This study is a Bidirectional Mendelian Randomization Study, which used MR to analyze the two-way causal relationship between insulin related traits and the risk of ovarian cancer. We found that the insulin secretion rate has a two-way causal relationship with ovarian cancer, which is rarely reported.

Insulin is an important hormone in mammalian homeostasis regulation, which regulates metabolism together with glucagon antagonism. The insulin secretion rate provides an estimate of the insulin secretion rate before hepatic insulin clearance (25). The main physiological stimulation of insulin secretion is the increase of circulating glucose concentration in the postprandial state. Impaired insulin secretion is often associated with high body mass index, and a large number of statistics have proved the association between overweight and ovarian cancer (15, 26). As mentioned in the introduction of this article, impaired insulin status is associated with the risk or survival of many cancers.

By analyzing the MR results in this paper, we could easily find that impaired insulin secretion was associated with an increased incidence of ovarian cancer. We will analyze the influence of insulin secretion on ovarian cancer from the following aspects. First, from the perspective of insulin and tumor cell energetics, compared with healthy cells, ovarian cancer tumor cells have a huge energy demand to support the abnormal proliferation and metastasis of tumors. Compared with normal human cells, tumor cells tend to change their metabolic mode, such as the transformation of primary glucose utilization pathway from oxidative phosphorylation to glycolysis, namely Warburg effect (27). Insulin also controls systemic and intracellular metabolism through substrate (glucose) distribution (28). However, tumors have changes in PI3K mTOR signaling pathway, and mTOR also changes the availability of glucose in tumor cells by regulating glucose uptake and glycogen decomposition (29). At the same time, anti-tumor drugs targeting systemic glucose homeostasis and tumor growth regulation have also entered the clinical trial stage (30). Second, impaired insulin secretion is associated with glucagon. Hyperinsulinemia is associated with the increased risk of breast cancer (31), endometrial cancer (32), ovarian cancer (33) and prostate cancer (34), and is closely related to the increased mortality of pancreatic cancer and breast cancer (35, 36), and some studies indicate that glucagon increases the overall mortality of cancer (37). However, it should be noted that many studies have pointed out that the postmenopausal serum insulin level is not related or is very weak to ovarian cancer after adjustment and correction (33, 38), which is consistent with the negative results of this study. However, we found that the significant results are insulin secretion rather than simple serum levels. At the same time, there is no denying that insulin and glucagon, which are hormones, are closely related to lipid peroxidation and metabolism (39), fibroblast growth factor receptor-1 (40), and inflammatory cytokines (41), and these factors undoubtedly play a key role in the progress of cancer. Third, the repeated mention of obesity or overweight is undoubtedly related to impaired insulin secretion. Ovarian cancer cells use fat cells as a source of energy for growth and migration (42). At the same time, as metabolic disorders, their internal metabolism affects each other. Because of changes in lifestyle factors, the prevalence of metabolic disorders is increasing year by year worldwide, just as obesity, type II diabetes and metabolic syndrome are all associated with ovarian cancer (43–46). A recent meta-analysis (47) showed that the risk of diabetes and OC was weak but still related, and many studies had many bias or confounding factors. It has also been pointed out that despite normal BMI, people with unhealthy metabolism or central obesity have a higher risk of cancer (48). Fourth, insulin is also related to immunity. Insulin (49) is related to regulating different immune phenotypes and responses, and the expression of insulin receptors on T cells, B cells and macrophages proves this view (50).

At the same time, another study showed that the existence of ovarian cancer was related to insulin secretion. The mechanism involved in this is very complex, because the metabolism of tumor variant fish is very complex. We speculate that the anaerobic glycolysis of tumors occupies the main form of metabolism, and pentose phosphate shunt pathway and its nucleotide products (51) play a certain role in the regulation of insulin secretion. Among them, glucose-6-phosphate dehydrogenase (G6PDH) (52) can explain the impairment of insulin secretion by islet cells through the impairment of NADPH production, and the 6-phosphogluconate dehydrogenase (6PGDH) (53) negative impact is attributed to the accumulation of intermediate metabolites of this pathway, leading to the activation of extracellular regulated kinase (ERK). Currently, it is known that ERK (53) can promote insulin transcription in response to acute signals, but its continuous activation may lead to β Cell dysfunction and apoptosis.

These studies have many defects and deficiencies, as follows: (1) Avoiding the pleiotropy of SNPs selected as instrumental variables is an important principle to ensure the accuracy of MR analysis. Usually, MR Egger intercept and MR-PRESSO methods are used to detect horizontal pleiotropy to reduce bias, but the method is not absolute for detection of pleiotropy. The MR analysis results of this study did not find heterogeneity and level pleiotropy, which proved the robustness of the results, but still could not completely rule out the interference of potential pleiotropy. This limitation is due to the existing analysis methods, and there are also some works (54) exploring other multiple validity testing methods. (2) The Insulin related Trains included in this study may lack some indicators. When selecting indicators, we selected open and common indicators. For the selection of databases, we also selected databases based on the same species, recent time and large number of people. However, this may not fully represent the function and release of insulin. We try to avoid these limitations, but it is undeniable that they may still exist. (3) There is also the race problem in the database. In order to control the same race, we try to select European samples, which will undoubtedly affect the conclusion to be extended to other colored people. (4) The sample size of some indicators may not be enough to avoid bias, which is also caused by database restrictions.

## 5 Conclusion

Through the Bidirectional Mendelian Randomization analysis, we obtained the two-way causal relationship between the insulin secret rate and ovarian cancer, that is, the reduction of the insulin secret rate is related to the risk of ovarian cancer, and the occurrence of ovarian cancer also has an impact on the insulin secret rate. When this research needs large sample data research in the real world, we hope to have research to further verify this conclusion.

## Data availability statement

The original contributions presented in the study are included in the article/**Supplementary Material**. Further inquiries can be directed to the corresponding authors.

## Ethics statement

Ethical review and approval was not required for the study on human participants in accordance with the local legislation and institutional requirements. Written informed consent for participation was not required for this study in accordance with the national legislation and the institutional requirements.

## Author contributions

XW wrote the main manuscript. JL and LC developed the model, WL and JS collected and analyzed the data. ZY reviewed and revised the manuscript. ZW and HL designed the study. Other personnel participate in discussion and article revision. All authors contributed to the article and approved the submitted version.
