# Supplementary figures and images for: Bidirectional Mendelian randomization study of insulin-related traits and risk of ovarian cancer
Source: Front Endocrinol (Lausanne). 2023 Mar 1;14:1131767. doi: 10.3389/fendo.2023.1131767 (PMC10014907; doi:10.3389/fendo.2023.1131767)

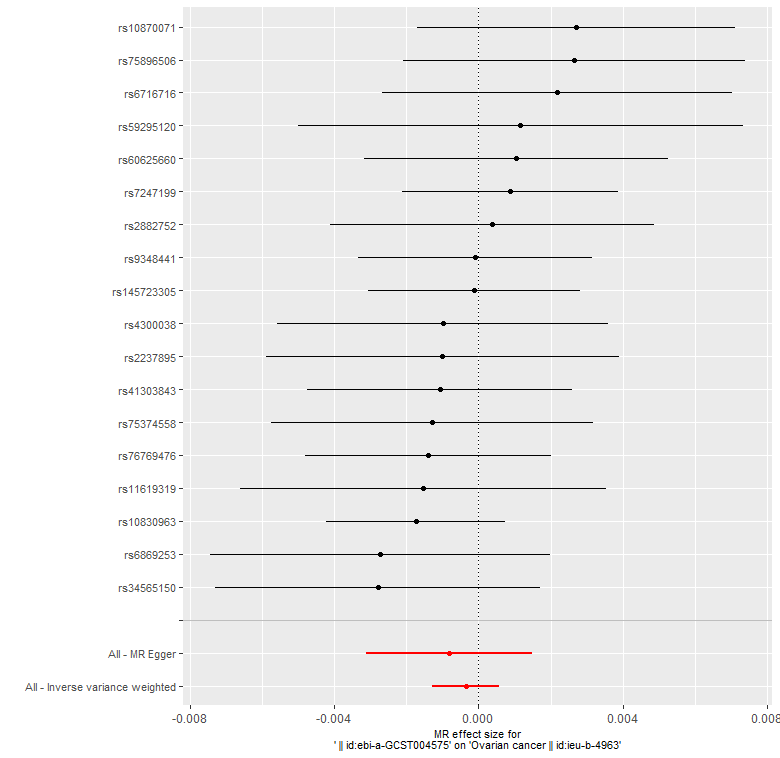

Supplement: Supplementary file 3 [file Image_1.png]

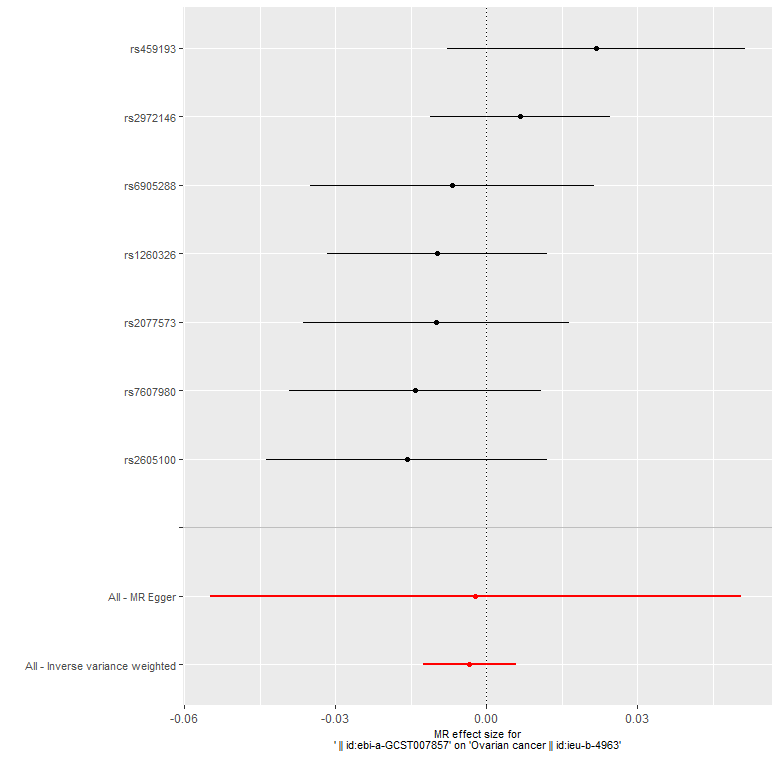

Supplement: Supplementary file 4 [file Image_2.png]

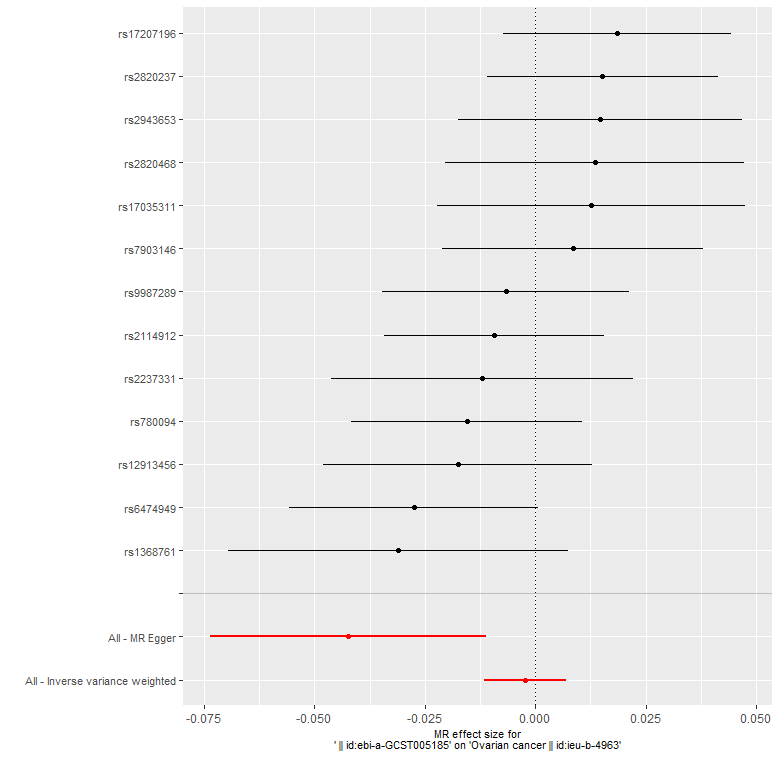

Supplement: Supplementary file 5 [file Image_3.png]

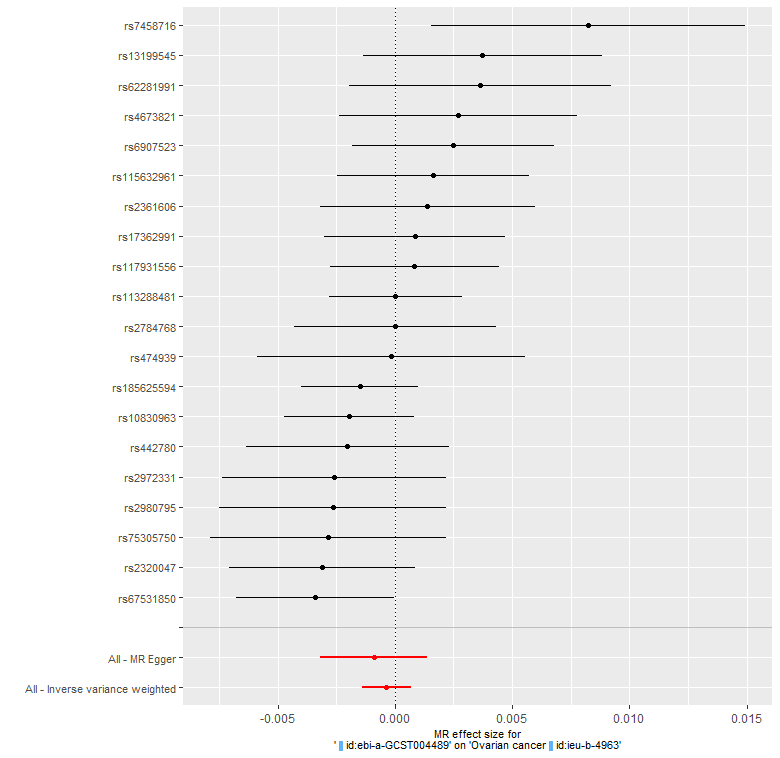

Supplement: Supplementary file 6 [file Image_4.png]

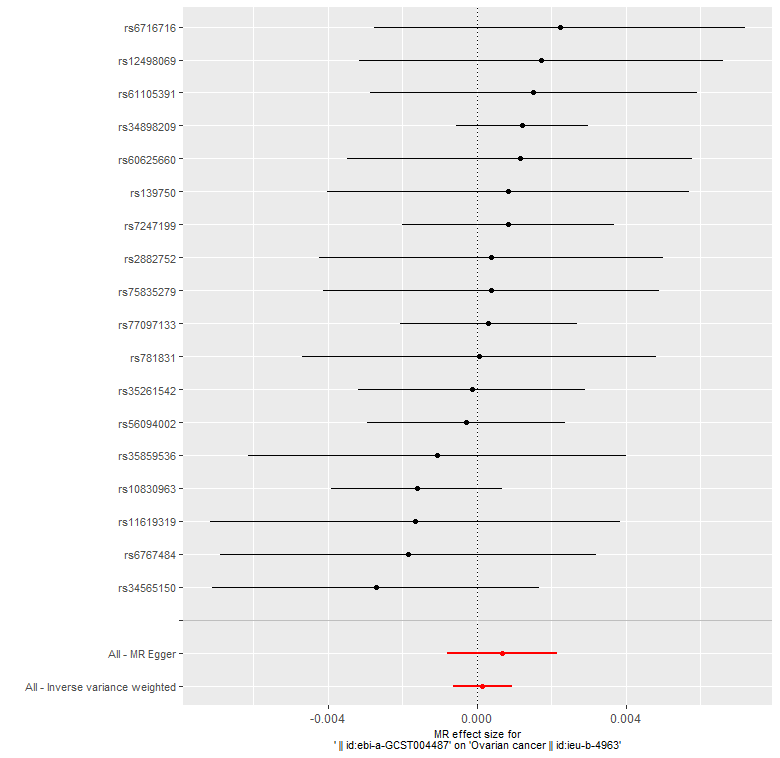

Supplement: Supplementary file 7 [file Image_5.png]

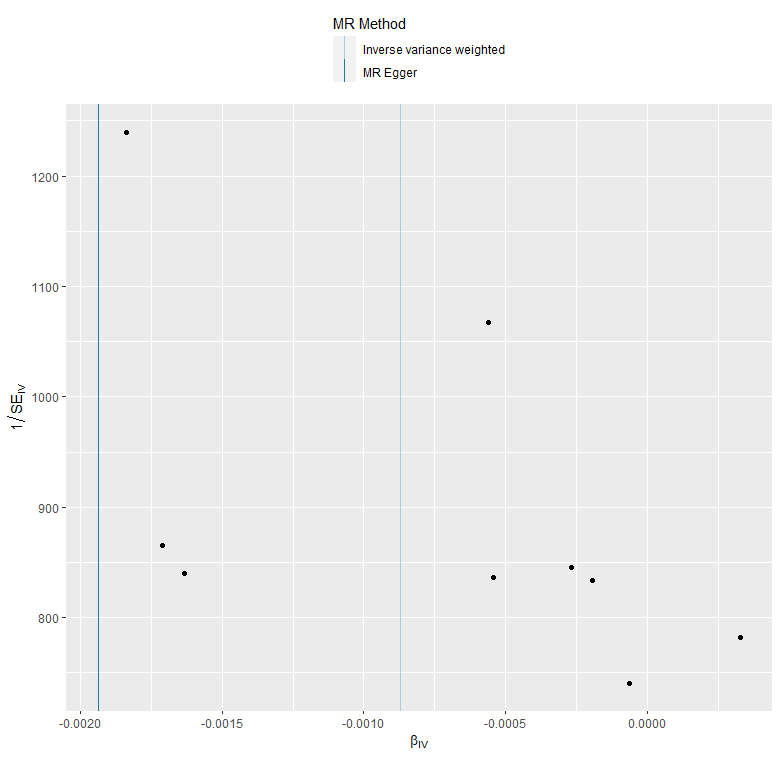

Supplement: Supplementary file 8 [file Image_6.png]

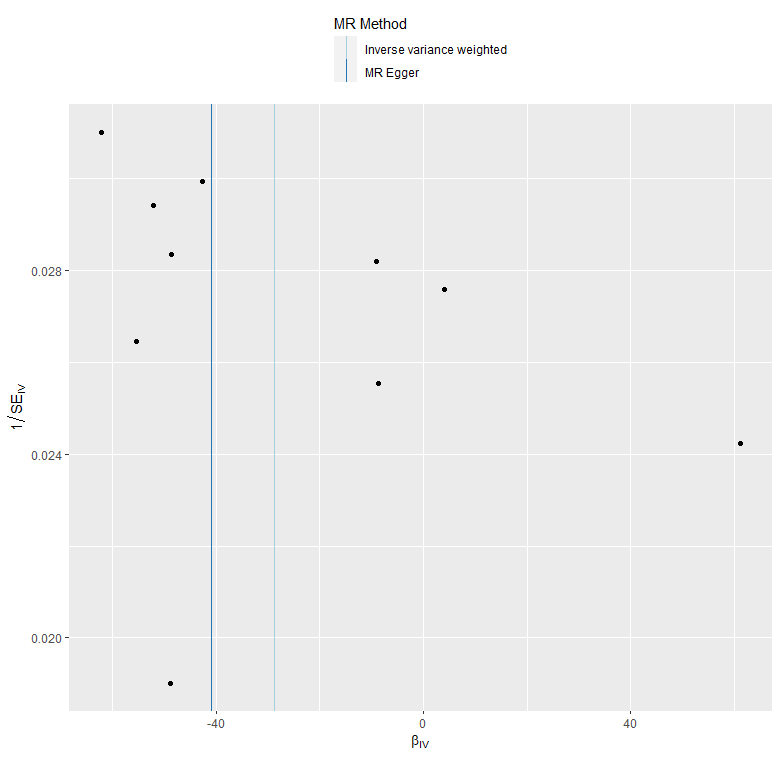

Supplement: Supplementary file 9 [file Image_7.png]
